# Anatomical observation and transcriptome analysis of branch-twisted mutations in Chinese jujube

**DOI:** 10.1186/s12864-023-09572-2

**Published:** 2023-08-29

**Authors:** Zhi Luo, Mengjiao Gao, Xuan Zhao, Lihu Wang, Zhiguo Liu, Lixin Wang, Lili Wang, Jin Zhao, Jiurui Wang, Mengjun Liu

**Affiliations:** 1 College of Horticulture, Hebei Agricultural University, Baoding, 071001 China; 2 Research Center of Chinese Jujube, College of Horticulture, Hebei Agricultural University, Baoding, 071001 China; 3 College of Forestry, Hebei Agricultural University, Baoding, 071001 China; 4 College of Life Science, Hebei Agricultural University, Baoding, 071001 China; 5 School of Landscape and Ecological Engineering, Hebei University of Engineering, Handan, 056038 China

**Keywords:** Chinese jujube, Twisted branches, Anatomy structure, *ZjTBL43*

## Abstract

**Background:**

Plant organs grow in a certain direction and organ twisted growth, a rare and distinctive trait, is associated with internal structure changes and special genes. The twisted branch mutant of Chinese jujube jujube, an important fruit tree native to China and introduced to nearly 50 countries, provides new typical materials for exploration of plant twisted growth.

**Results:**

In this study, the cytological characteristics and related genes of twisted branches in Chinese jujube were revealed by microscopy observation and transcriptome analysis. The unique coexistence of primary and secondary structures appeared in the twisted parts of branches, and special structures such as collateral bundle, cortical bundles, and internal phloem were formed. Ninety differentially expressed genes of ‘Dongzao’ and its twisted mutant were observed, in which *ZjTBL43*, *ZjFLA11*, *ZjFLA12* and *ZjIQD1* were selected as candidate genes. *ZjTBL43* was homologous to *AtTBL43* in *Arabidopsis*, which was involved in the synthesis and deposition of cellular secondary wall cellulose. The *attbl43* mutant showed significant inflorescence stem bending growth. The transgenic lines of *attbl43* with overexpression of *ZjTBL43* were phenotypically normal.The branch twisted growth may be caused by mutations in *ZjTBL43* in Chinese jujube. *AtIQD10*, *AtFLA11* and *AtFLA12* were homologous to *ZjIQD1*, *ZjFLA11* and *ZjFLA12*, respectively. However, the phenotype of their function defect mutants was normal.

**Conclusion:**

In summary, these findings will provide new insights into the plant organ twisted growth and a reference for investigation of controlling mechanisms of plant growth direction.

**Supplementary Information:**

The online version contains supplementary material available at 10.1186/s12864-023-09572-2.

## Background

Plant organs usually grow in a certain direction and have specific forms, such as roots growing down and petals flattening [1]. Organ twisting is a rare phenomenon, which has peculiar shape and high ornamental value. Organ twist traits are primarily related to genetic factors [2, 3]. There are typical examples of twisted organ growth in some plants, such as curved plum, corkscrew willow, ‘Dalilongzao’ jujube, the twisted bud mutant ‘Dongzao’ in woody plants [2–6], dodder *Cuscuta spp*, honeysuckle *Lonicera*, and morning glory (*Ipomoea nil*) in vines [7–10].

The normal and twisted growth of plants showed significant differences not only in organ morphology but also in the internal structure of organs. In *Arabidopsis*, the hypocotyl surface is rough, hypocotyl epidermal cells are distorted [11], the petals are left-handed twisted, and the petal epidermal cells are nanotube shaped and left-hand twisted [12]. The cotyledons and petals of tomato mutants are right-handed and twisted forms, with twisted internal cells [13]. In addition, willow trees show twist and variation, with smaller branch phloem fiber clusters and later lignification development [4].

The molecular mechanism of plant twisted growth has been gradually revealed in the past two decades. The majority of plant organ twisted growth is associated with microtubule changes, and mutations in microtubule-related genes cause twisted growth on the left or right hand [14–28]. The *SPIRAL1* and *SPIRAL2* genes can cause twisted growth in *Arabidopsis* by affecting microtubules [14]. The loss of the *SPIRAL1* and *SPIRAL2* genes leads to the growth of the right-handed helix, which causes twisting of the right-hand form in the root, stem and hypocotyl. The latter also causes twisting of the right-hand type in the petiole and petals. The *SPIRAL1* and *SPIRAL2* double mutants have a better phenotype than the single gene mutant, and the addition of the microtubule interaction drug propylamamide or taxinol can lead to the growth of the left-handed helix in wild-type *A. thaliana*. According to previous studies, a small number of plants have organ twisted growth related to genes that are associated with cell wall components [29]. The *Arabidopsis* mutant (known as *rhm1*), defective in UDP-L-rhamnose synthase, showed an altered pectin composition and left-handed petals. The pectin composition of the *Arabidopsis* mutant *mur8* is reduced, resulting in left-handed twisted helical growth in the root [12].Therefore, the plant twisted growth is a result of changes of microtubule regulation or wall components, in which several genes play key roles.

Chinese jujube is an important fruit tree native to China that has been introduced to nearly 50 countries on five continents. In a previous report, we obtained the typical twisted-branch mutants in ‘Dongzao’, a main cultivar in Chinese jujube [6]. In this paper, we performed microscopic observations and transcriptome analysis and found the abnormal tissue structure of twisted branches and the gene related to the distorted variation of branches in Chinese jujube. The results can have theoretical and application value for revealing the mechanisms of branch distortion and regulating the plant growth direction.

## Results

### Anatomical features of the twisted branches

**Fig. 1:**
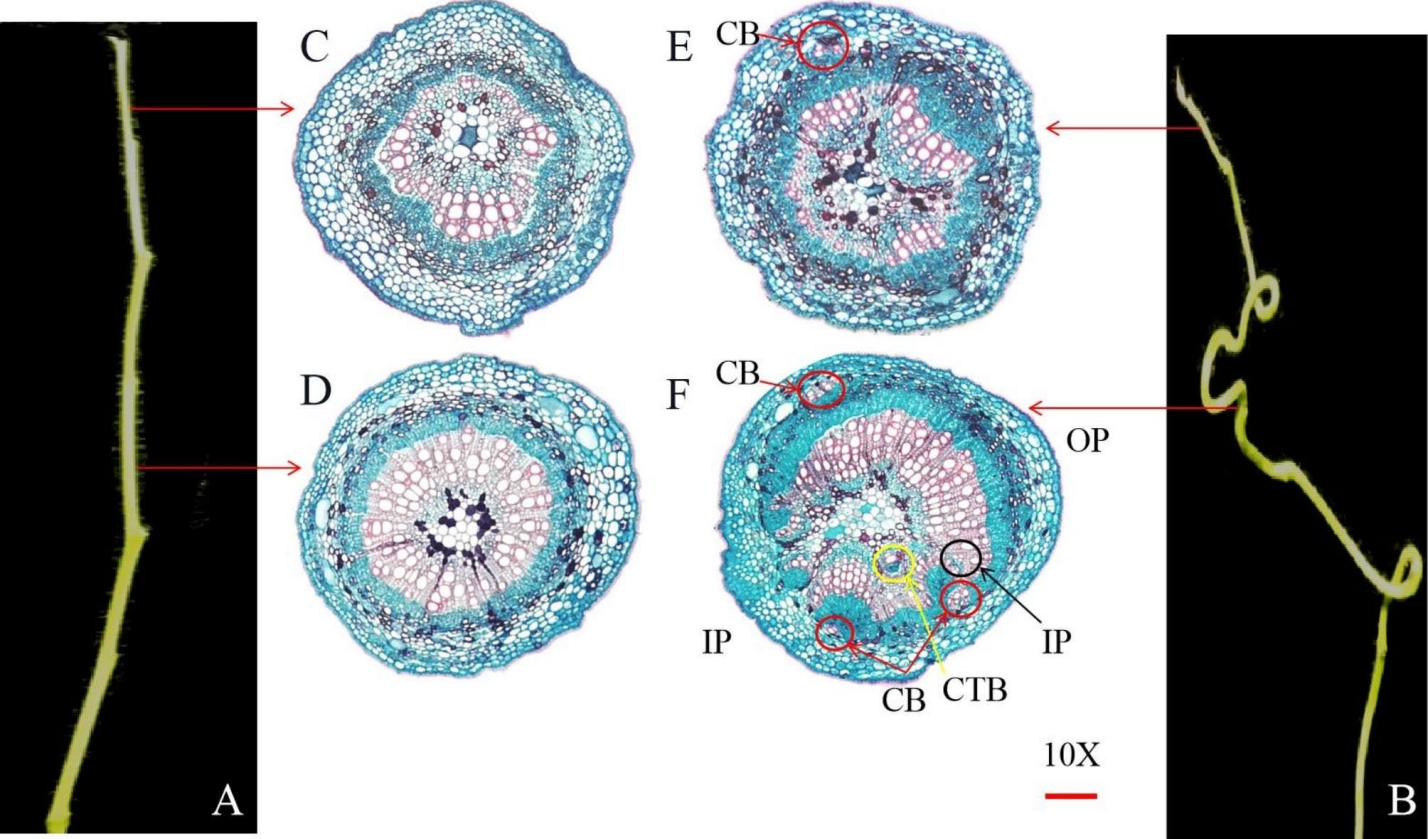
Morphology and anatomical structure of bearing shoot in ‘Dongzao’ and its twisted branch mutant **A.** ‘Dongzao’ bearing shoot **B.** Twisted mutant bearing shoot. **C**, **D** The anatomy structure of ‘Dongzao’ bearing shoot. E, F Anatomical structural of the twisted bearing shoot. The yellow circle is the collateral bundle CTB, the black circle is the internal phloem IP, the red circle is the cortical bundle CB, IP is the inside part site, and OP is the outside part site

The microscopic observation of the bearing shoots without leaves (Fig. 1A, B) showed that the tissue structure of the bearing shoots in ‘Dongzao’ was significantly different from that in its twisted branch mutant. The primary xylem, cambium and primary phloem of ‘Dongzao’ were clearly layered (Fig. 1C, D). However, the tissue structure of the twisted bud branches was abnormal, and cortical bundle (CB) appeared around the primary phloem (Fig. 1E). The inside part of twisted branches (Fig. 1 IP) developed slowly, in which the xylem, cambium and phloem differentiation were not obvious, and the cells were small, similar to the primary structure. The outside part showed normal development (Fig. 1 OP) and belonged to typical secondary structure, in which the xylem, cambium and phloem differentiation were relatively obvious and the cells were regular and large. The coexistence of primary and secondary structures appeared in the twisted parts. In addition, the special structure of collateral bundle (CTB), cortical bundles (CB) and internal phloem (IP) appeared (Fig. 1F).

In the bearing shoots of twisted bud mutant, abnormal cortical bundles appeared first, followed by the collateral bundle and internal phloem, and the coexistence of primary and secondary structures. The anatomical structure of the inner and outer sides of the bearing shoot showed obvious growth difference, that caused twisted growth of shoots.

### Comparison of the transcriptome of ‘Dongzao’ and its twisted mutant

The sequencing was filtered to obtain a total of 42.10 Gb of clean data, where the clean data for each sample were approximately 6.22 Gb. In addition, approximately 85% of reads matched the jujube reference genome database (Table S1). A total of 31,971 expressed genes were detected, including 30,011 sequences matching the known genome.

**Fig. 2:**
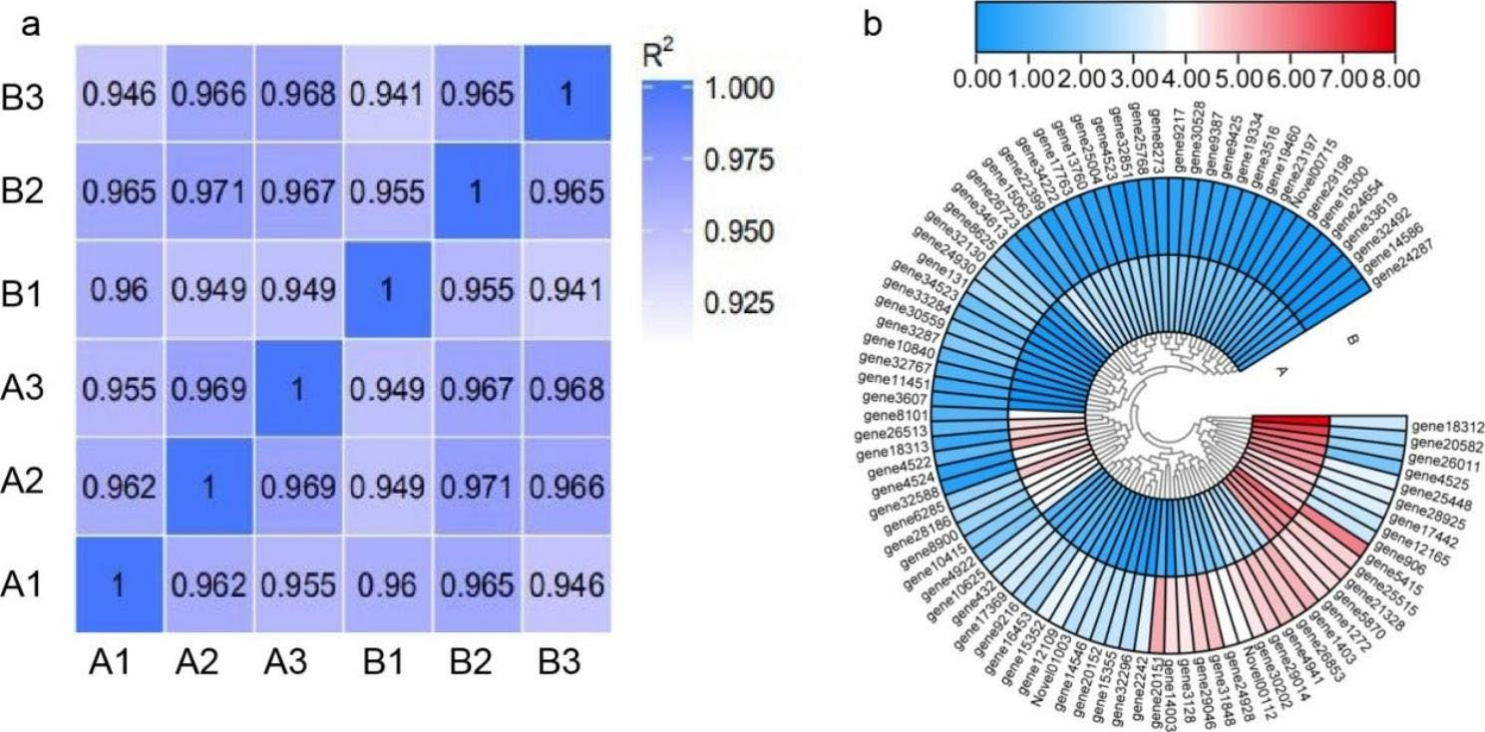
Transcriptome sequencing of bearing shoot in‘Dongzao’ and its twisted branch mutant. **(A)** Correlation between samples. **(B)** Heatmap analysis of differential genes **(A)** ‘Dongzao’ **(B)** Twisted branch mutant

By sample RNA-Seq correlation analysis, the R^2^ of A1, A2 and A3 was at least 0.955 between the replicates of ordinary ‘Dongzao’ samples, and the R^2^ of B1, B2 and B3 was at least 0.941. The lowest R2 between samples for A1 and B3 was 0.946 (Fig. 2a). The high similarity of expression patterns between samples indicated the high reliability of our sequencing data.

There were obvious differences in gene expression between ‘Dongzao’ and its branch-twisted mutant, indicating that branch twisting was regulated by differentially expressed genes (DEGs). To better understand the differentially expressed genes, 90 differentially expressed genes between ‘Dongzao’ and its branch-twisted mutant were identified using DESeq2 analysis (Fig. 2b, Table S2).

### Functional analysis of differentially expressed genes

**Fig. 3:**
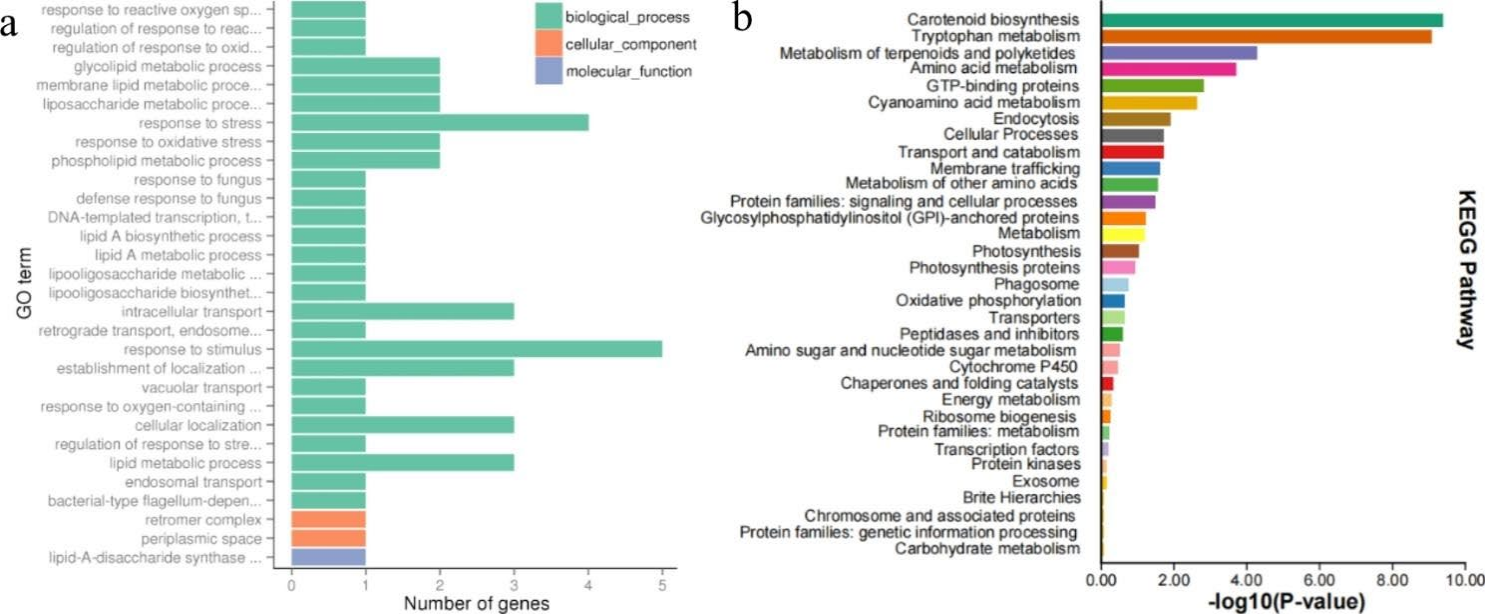
Histogram of differential gene (GO) enrichment and pathway (Kyoto Encyclopedia of Genes and Genomes KEGG) classification. a.GO enrichment analysis b. KEGG pathway analysis

To gain insight into the function of the differentially expressed genes, we performed GO annotation and enrichment analysis for all the differentially expressed genes. As shown in Fig. 3a and Table S3a, differentially expressed genes were divided into three groups by molecular function, cellular components and biological processes. In biological processes, most of the differentially expressed genes were mainly involved in the stress response (GO: 0006950), stimulus response (GO: 0050896), lipid metabolic process (GO: 0006629) and metabolic process of phosphate compounds (GO: 0006796). During the molecular process category, catalytic activity (GO: 0003824), binding (GO: 0005488), and peptidase activity (GO: 0070011) were significantly enriched. In addition, further KEGG analysis showed that the differential genes were involved in the pathway of carotenoid biosynthesis, tryptophan metabolism, terpenoids and polyketides metabolism, transport and catabolism (Fig. 3b, Table S3b).

### qRT‒PCR validation of differentially expressed genes between ‘Dongzao’ and its twisted mutant

**Fig. 4:**
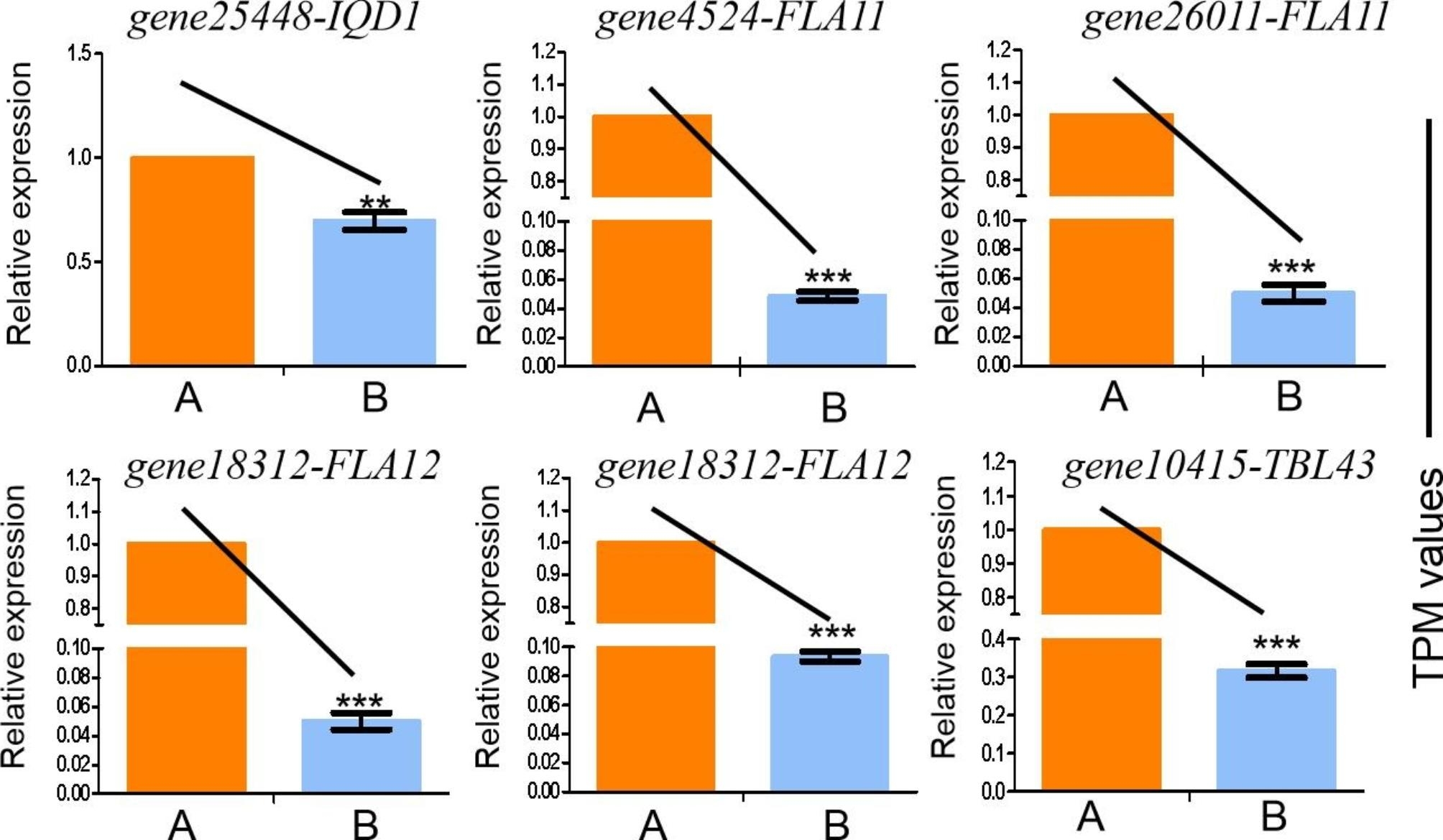
Verification of differentially expressed genes by qRT‒PCR. B. Comparison of the expression levels of differentially expressed genes and TPM values. **(A)** ‘Dongzao’ **(B)** Twisted branch mutant

The previous reports showed genes related to microtubules or cell walls were involved in plant distorted growth. To verify the reliability of the transcriptomic data. the differential genes related to the microtubule and cell wall metabolic pathways were selected for qRT‒PCR analysis. The results showed that the expression pattern of the genes was consistent with the transcripts per million reads(TPM)(Fig. 4). For example, *ZjFLA11* and *ZjFLA12* (Fasciclin-like arabinogalactan) function were arabinogalactose in the constituent pectin components, and *ZjIQD1* (Ca^2+^)-dependent calmodulin-binding protein) binded to SPR2 to maintain the stability of microtubules. *ZjTBL43* (TRICHOME BIREFRINGENCE-LIKE) was involved in the synthesis and deposition of secondary wall cellulose. It was found that these differential genes related to microtubules and cell wall metabolism were downregulated in the twisted branch.

### Functional validation of the candidate gene IQD

**Fig. 5:**
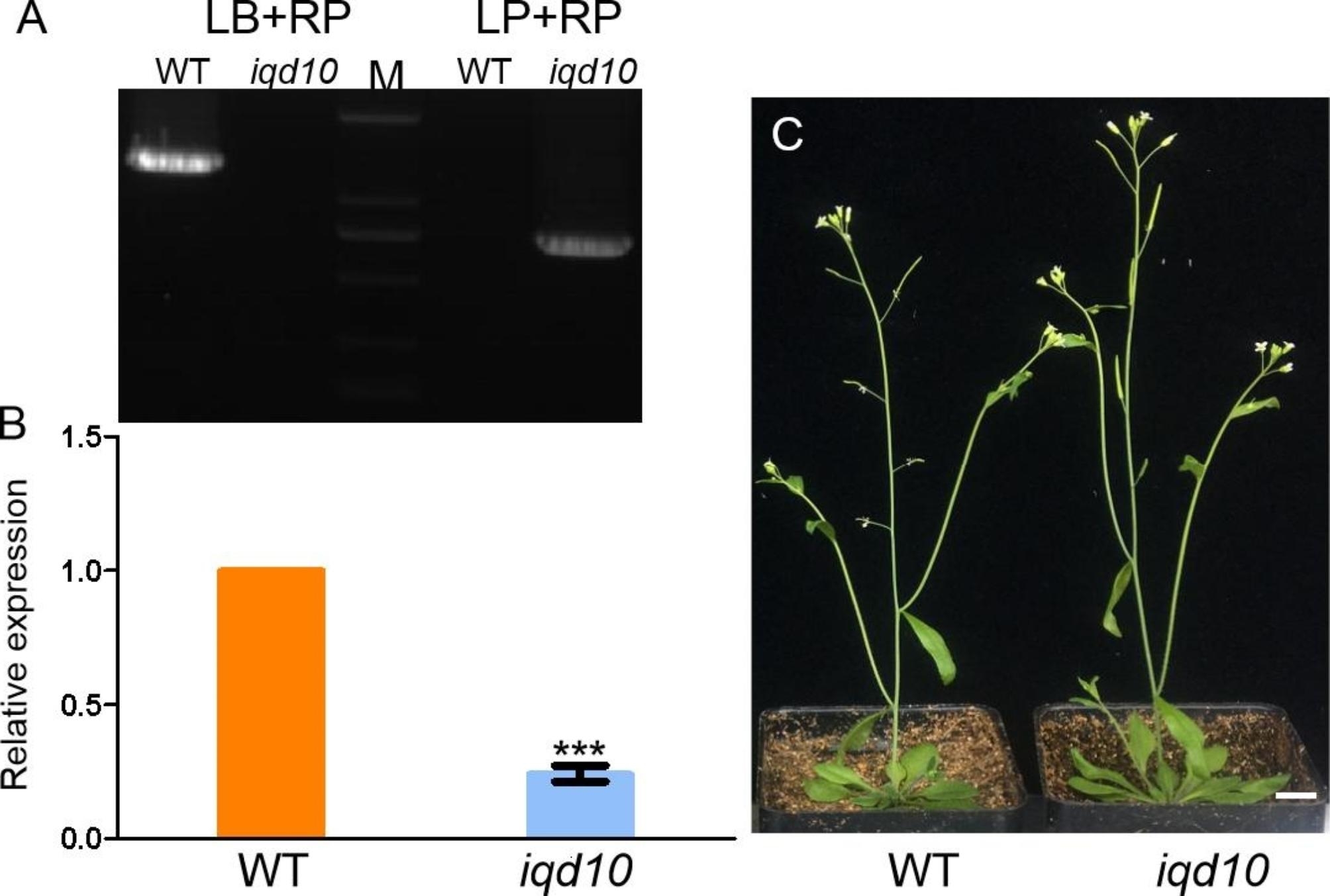
Analysis of *atiqd10* function. **(A)** RT‒PCR of wild-type *Arabidopsis* and *atiqd10* plants. **(B)** qRT‒PCR detection of wild-type *Arabidopsis* and *atiqd10* plants. **(C)** Wild-type and *atiqd10* plants with phenotype. Bar 1 cm. M represents the marker

Based on the transcriptome analysis reported by previous studies and this study, most of the candidate genes *IQD1* (Ca^2+^-dependent calmodulin-binding protein) considered to be associated with microtubules were first selected. Through amino acid sequence alignment, the homologous gene of the *ZjIQD1* gene is *AtIQD10* (*AT3G15050*). The *atiqd10* (SALK_111401C) mutant was purchased from AraShare, and RT‒PCR identified the *atiqd10* plants as homozygous mutants (Fig. 5A).The full-length gels were presented in Supplementary S4. Quantitative real-time testing showed that all *atiqd10* plants had low expression compared to wild-type Columbia *A. thaliana* (Fig. 5B). However, *atiqd10* plants showed normal growth and no twisted organ growth phenotype (Fig. 5C). The above results indicated that the microtubule-associated gene *IQD1* cannot cause distorted growth in plants.

### Functional validation of ZjTBL 43 ***ZjFLA11 and ZjFLA12***

**Fig. 6:**
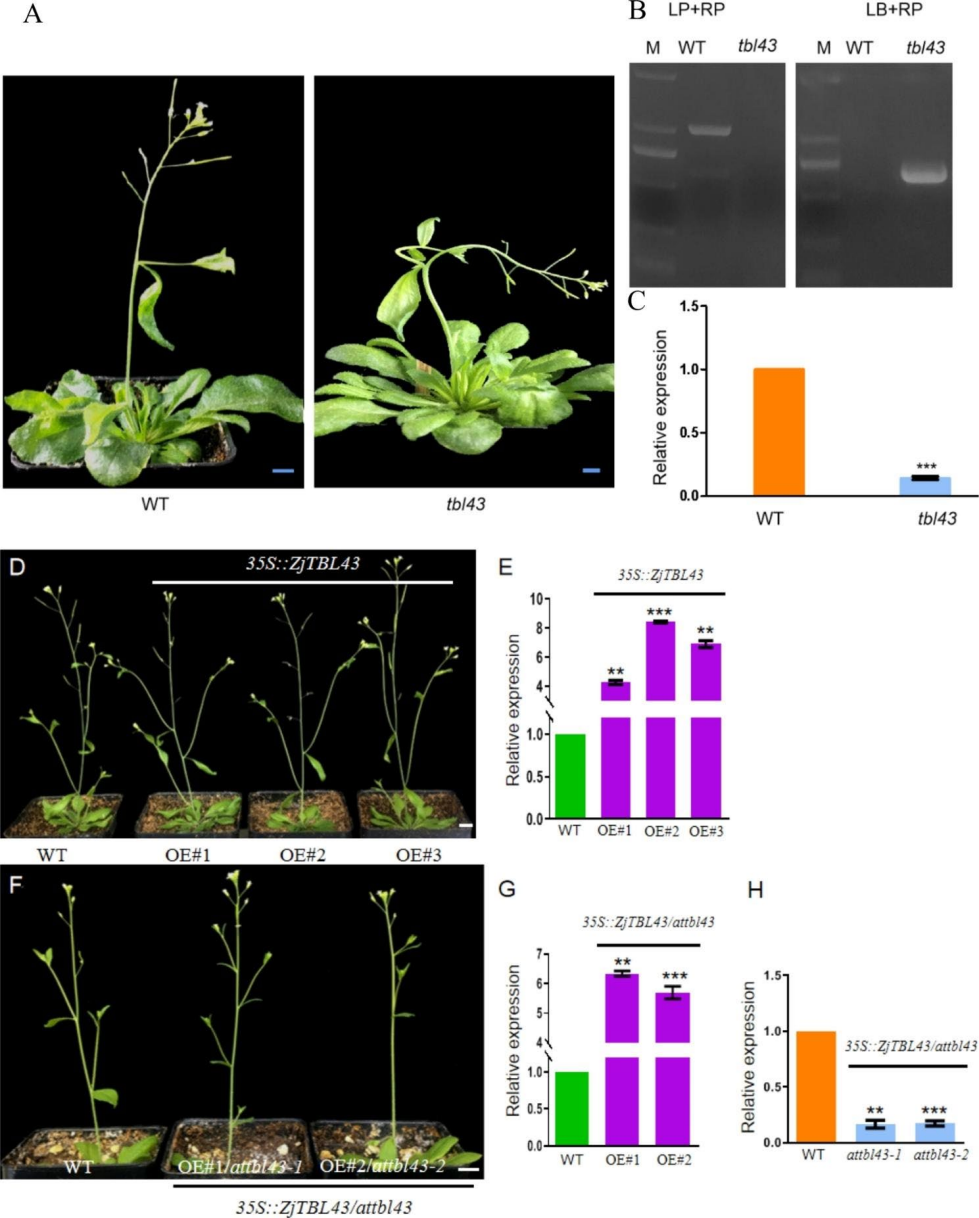
Analysis of *attbl43* function. **(A)** wild-type *Arabidopsis* and *attbl43* plants. **(B)** RT‒PCR detection of wild-type *Arabidopsis* and *attbl43* plants. **(C)** qRT‒PCR detection of wild-type *Arabidopsis* and *attbl43* plants. **(D)** Overexpressing the *ZjTBL43* gene in the wild-type Arabidopsis. **(E)** Expression of the transgenic *ZjTBL43* line. **(F)** Overexpression of the *ZjTBL43* gene in the mutant *attbl43*. **(G)***ZjTBL43* expression in the *35 S:: ZjTBL43/attbl43* line. H. expression of *attbl43* in the *35 S:: ZjTBL43/attbl43* line. Bar 1 cm. M represents the marker

Except for the microtubule-related gene *IQD 1*,cell wall-related genes including *ZjFLA11*, *ZjFLA12 and ZjTBL43* served as the second class of target genes. By alignment of the amino acid sequence of *Arabidopsis*, the *Arabidopsis* homologous genes corresponding to the *ZjFLA11*, *ZjFLA12* and *ZjTBL43* are *AtFLA11* (*AT5G03170*), *AtFLA12* (*AT5G60490*) and *AtTBL43* (*AT2G30900*), respectively. The *atfla11* (WiscDsLoxHs052_12C), *atfla12* (SALK_206269 C), and *attbl43* (SALK_047584 C) mutants were purchased on AraShare, and the *atfla11* and *atfla12* plants were phenotypically normal, indicating that the Chinese jujube branch twisting was not caused by the *ZjFLA11* and *ZjFLA12* genes. *Attbl43* plants were identified as homozygous mutants by RT‒PCR (Fig. 6A). The full-length gels were presented in Supplementary S5. By real-time quantitative PCR, *TBL43* genes were expressed at low levels in *attbl43* plants compared with wild-type *Arabidopsis* (Fig. 6B), and *attbl43* plants showed curved growth (Fig. 6C). These results showed that *TBL 43* was downregulated and inflorescence stem bend growth in *attbl43* plants. Furthermore, transgenic Arabidopsis lines incorporating *ZjTBL43* were phenotypically normal (Fig. 6D E). The *35 S:: ZjTBL43/attbl43* (overexpressing *ZjTBL43* in *attbl43* mutant plants) lines showed an upright growth morphology (Fig. 6F G H). This indicated that the *ZjTBL43* gene can compensate for the loss of *attbl43*.

## Discussion

Compared with normal growing plant organs, the internal structure of twisted plant organs shows abnormal cell morphology and abnormalities in the phloem, xylem, and cambium [4, 11–13]. The collateral bundle is the vascular bundle located in the pulp of the dicotyledonous stem. Cortical bundles are found in some dicotyledon plants that are isolated in the vascular column and that are consistently preserved in the cortex. In some dicotyledonous plants, there are bundles or continuous rotational phloem around the inner margin or pulp of the xylem, namely, the internal phloem [30]. Whether the abnormal structure of the collateral bundle, cortical bundle and internal phloem is related to plant twisted growth has not been reported. In this study, we observed for the first time the rare phenomena of the collateral bundle, cortical bundle and internal phloem of bearing shoots, and the coexistence of primary structures and secondary structures appeared. The internal structure of the twisted branches was obviously different. There was no coexistence of primary and secondary structures in the curved branches of ‘Longzhuazao’, but other special structures appeared, such as the auxillary bundle, interxylary phloem and medullary bundle [31]. The collateral bundle, cortical bundle and internal phloem are abnormal primary structures, while the auxillary bundle, interxylary phloem and medullary bundle are abnormal secondary structures [30, 31]. The differences of internal structure between the branch-twisted mutant of ‘Dongzao’ and ‘Longzhuazao’ indicated that there may be different mechanisms controlling their twisted branch development.

The changes in microtubule composition and mutations in microtubule-associated proteins can lead to twisted growth of plant organs [29]. IQD family members of microtubule-associated proteins, as different signal integrators, can connect auxin signals to the downstream microtubule regulator SPR2, while IQD proteins bind to SPR2, thus inhibiting the function of microtubule end stabilization [26, 27, 32]. In this study, the expression of microtubule-related gene *IQD1* was significantly downregulated in the twisted branch of ‘Dongzao’ mutant. We speculated that IQD1 gene might be associated with branch twisted growth. However, *Arabidopsis* plants without *IQD1 gene* function did not show distorted growth. It is possible that the *IQD1* genes found in this paper have gene redundancy in *Arabidopsis*, and further studies should be conducted to determine whether the presence of redundant *IQD1* genes can assist normal plant growth.

The microtubule component is currently the majority reported for plant twisted growth [15, 16, 20, 22, 29, 33]. In addition to microtubule-related genes, plant organ twist is associated with the cell wall component genes *SKU 5* and *RHM1* [12, 34]. In this study, the expression of *ZjTBL43, ZjFLA11*, and *ZjFLA12* was significantly different and related to cell wall components. Therefore, it was speculated that the *ZjTBL43, ZjFLA11* and *ZjFLA12* genes were associated with the branch distorted growth of Chinese jujube. Further tests showed that *ZjTBL43* was involved in twisted growth of branch. The low expression of *ZjTBL43* gene probably caused the changes of cell wall composition and cell growth, leading to the special tissue structure formatin and branch twisted growth.

## Conclusions

The unique coexistence of primary and secondary structures and special structures such as collateral bundle, cortical bundles, and internal phloem appeared in the twisted parts of Chinese jujube branches. The candidate genes *ZjTBL43*, *ZjFLA11*, *ZjFLA12* and *ZjIQD1* associated with twisted growth were selected from 90 genes obtained by transcriptome analysis. *ZjTBL43* was involved in the synthesis and deposition of cellular secondary wall cellulose. *ZjTBL43* was a homologous gene with *AtTBL43* and the function defect of *attbl43* in *Arabidopsis* showed stem bending growth. The transgenic lines of *attbl43* with overexpression of *ZjTBL43* were phenotypically normal. Furthermore, Although the genes *ZjIQD1*, *ZjFLA11* and *ZjFLA12* were differentially expressed in Chinese jujube, the function defect mutants of their homologous genes *atiqd10*, *atfla11* and *atfla12*, showed normal growth. The branch twisted growth may be caused by mutations in *ZjTBL43* in Chinese jujube. This study is valuable for deeply revealing the control mechanisms of branch distortion and plant growth direction.

## Materials and methods

### Plant materials and cultivation

‘Dongzao’ and its twisted branch mutant were used in this study. The scions from the twisted variant and ‘Dongzao’ tree were top-grafted on five-year-old healthy ‘Dongzao’ trees in 2014 at the same time [6]. *Arabidopsis thaliana* (Col-0) seeds were obtained from Xuan Zhao from China Agriculture University and sown in a soil medium matrix (peat:vermiculite = 1:1) under a 16 h light/8 h darkness photoperiod at 20 ± 2 °C and a relative humidity of 60 ± 5%.The *Arabidopsis iqd10*,*tbl43,fla11 and fla12* mutant was obtained from AraShare Science (https://www.arashare.cn/index/).

### Organizational observation

The bearing shoot of ‘Dongzao’ and its twisted branch mutant at the same development stage were taken as materials, and the tissue structure of the test materials was observed by paraffin section. The test material was first fixed with the FAA fixative (component 90% ethanol, 5% acetic acid, and 5% formalin) and subsequently dehydrated, paraffin-embedded, sectioned, stained, sealed, and air-dried for observation. Images of the tissue cell sections were analyzed using image software (ZP-1000 microscope, PuZhe, ShangHai, China).

### RNA extraction, library preparation and sequencing

RNA in bearing shoots of ‘Dongzao’ and its twisted mutant was extracted by an RNA rapid extraction kit (Tiangen Biotechnology, China). RNA quality was measured using a Nanodrop2000 (Thermo NanoDrop 2000, ShangHai, China) and 1% agarose gel electrophoresis. Then, RNA-seq transcription libraries were constructed and sequenced using the sample preparation kit. The library preparations were sequenced on an Illumina Hiseq platform and 125 bp/150 bp paired-end reads were generated. Reference genome and gene model annotation files were downloaded from the Ensembl Genomes website (https://www.ncbi.knlm.nih.gov/assembly/GCF_000826755.1). Original sequencing data uploaded to the NCBI-SRA database China National GeneBank DataBase (CNGBdb) (https://db.cngb.org/)(In Availability of data and materials).

### Transcriptome data analysis, differential gene identification and gene annotation

After adapter and low-quality sequence filtering of the transcriptome machine data, the read data were converted TPM. The differentially expressed genes were analyzed using TBtool Software [35]. The cloud platform was used to obtain gene annotations, and a heatmap was made using TBtools software.

### qRT‒PCR analysis

qRT‒PCR analysis was performed on a Bio-Rad real-time quantitative instrument. A real-time quantification kit (Tiangen Biotechnology, China) was used. The 20 µL reaction system contained 10 µL of 2 SYBR Premix, 10 µM of primers 0.4 µL each, 1 µL of diluted cDNA and 8.2 µL ddH_2_O. They were incubated at 94 °C for 15 min, followed by 15 s at 94 °C, 55 to 63 °C, and 15 s at 72 °C. *ZjActin* and *AtActin* were used as reference genes [36, 37], and gene expression was calculated using the 2^−ΔΔCT^ method [38]. The sequences of primers for qRT‒PCR are shown in Supplementary Table S6.

### Gene overexpression

The *ZjTBL43* gene primers were designed(Supplementary Table S6)and the *ZjTBL43* gene sequence was obtained by PCR amplification. A *35:: ZjTBL43* overexpression vector (pCambia1391) was constructed and transformed into Agrobacterium GV3101. Wild-type Arabidopsis and *attbl43* mutant were infected by floral dipping. The T1 transgenic lines were obtained by seedling screening with hygromycin.

### Data analysis

Data for each experiment were based on three biological replicates. Statistical analysis was performed using the EXCE T test, and the results are presented as the mean (M ± SE). There was a significant difference at *p* ≤ 0.05 (*p* < 0.05 *, *p* < 0.01 **, *p* < 0.001 ***).

### Electronic supplementary material

Below is the link to the electronic supplementary material.

Supplementary Material 1

Supplementary Material 2

Supplementary Material 3

## Data Availability

The data that support the findings of this study have been deposited into CNGB Sequence Archive (CNSA) [39] of China National GeneBank DataBase (CNGBdb) (https://db.cngb.org/) [40] with accession number CNP0004018 (https://db.cngb.org/search/project/CNP0004018/). *Arabidopsis thaliana* (Col-0) materials used in the experiment were supplied by Doctor. Xuan Zhao of Hebei Agricultural University, and this material was used with permission. The datasets supporting the conclusions of this study are included in the article and its additional files.

## Abbreviations

WT: *Arabidopsis thaliana* (Col-0)
qRT-PCR: Quantitative real-time PCR
