# Supplementary figures and images for: Anatomical observation and transcriptome analysis of branch-twisted mutations in Chinese jujube
Source: BMC Genomics. 2023 Aug 29;24:500. doi: 10.1186/s12864-023-09572-2 (PMC10466873; doi:10.1186/s12864-023-09572-2)

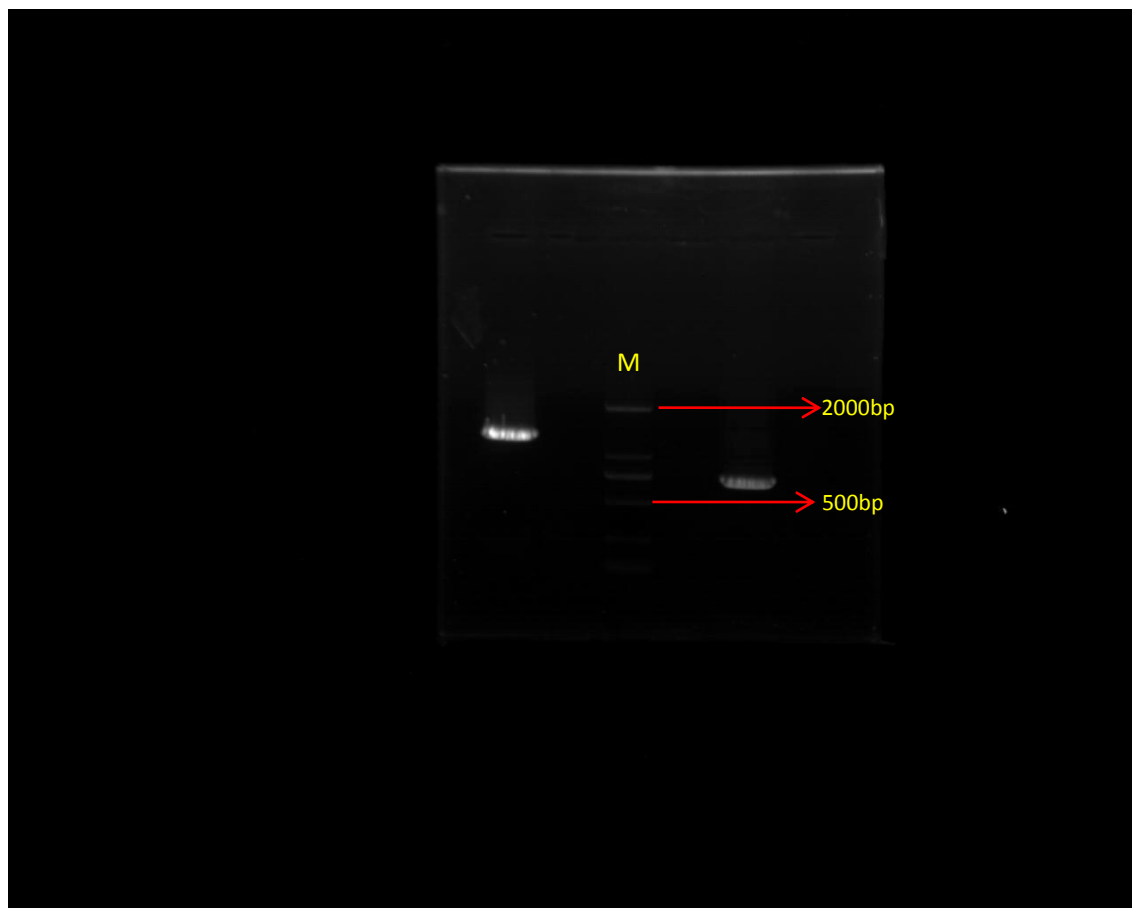

S4. *atigd10* full-length gels.

M represents Mark, and the six band from top to bottom is 2000,1000,750,500,250,100bp, respectively.

Supplement: Supplementary file 1 — Supplementary Material 1 [file 12864_2023_9572_MOESM1_ESM.pdf]
