# Supplementary material for: Anatomical observation and transcriptome analysis of branch-twisted mutations in Chinese jujube
Source: BMC Genomics. 2023 Aug 29;24:500. doi: 10.1186/s12864-023-09572-2 (PMC10466873; doi:10.1186/s12864-023-09572-2)
Supplement: Supplementary file 2 — Supplementary Material 2 [file 12864_2023_9572_MOESM2_ESM.pdf]

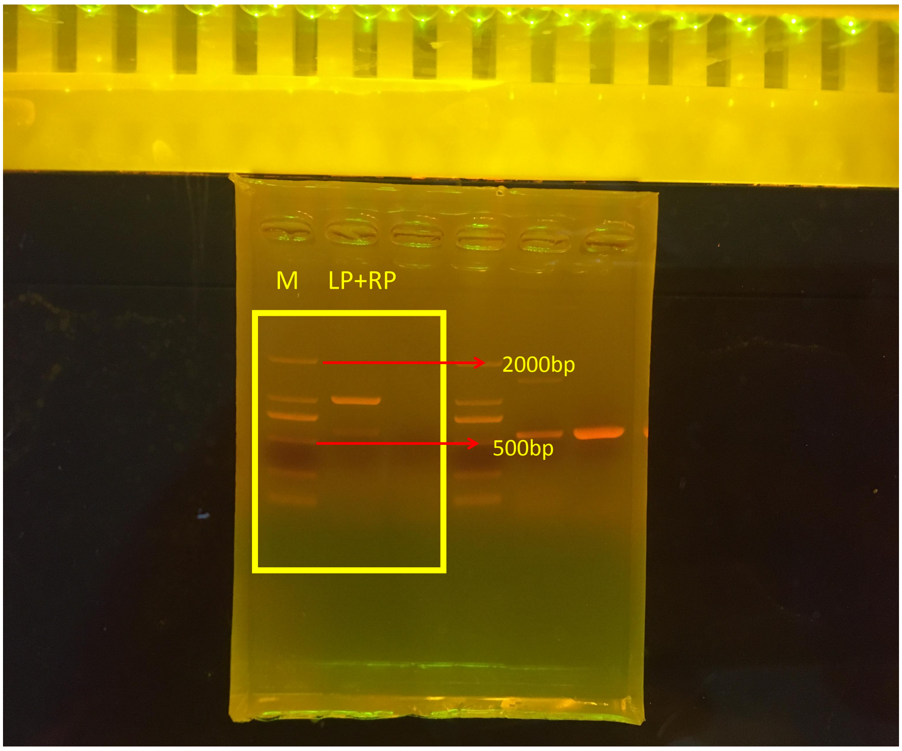

S5.*attbl43* half-length gels.

Note. The cropping figure（yellow box）used in the main body. M represents Mark, and the six band from top to bottom is 2000,1000,750,500,250,100bp, respectively.

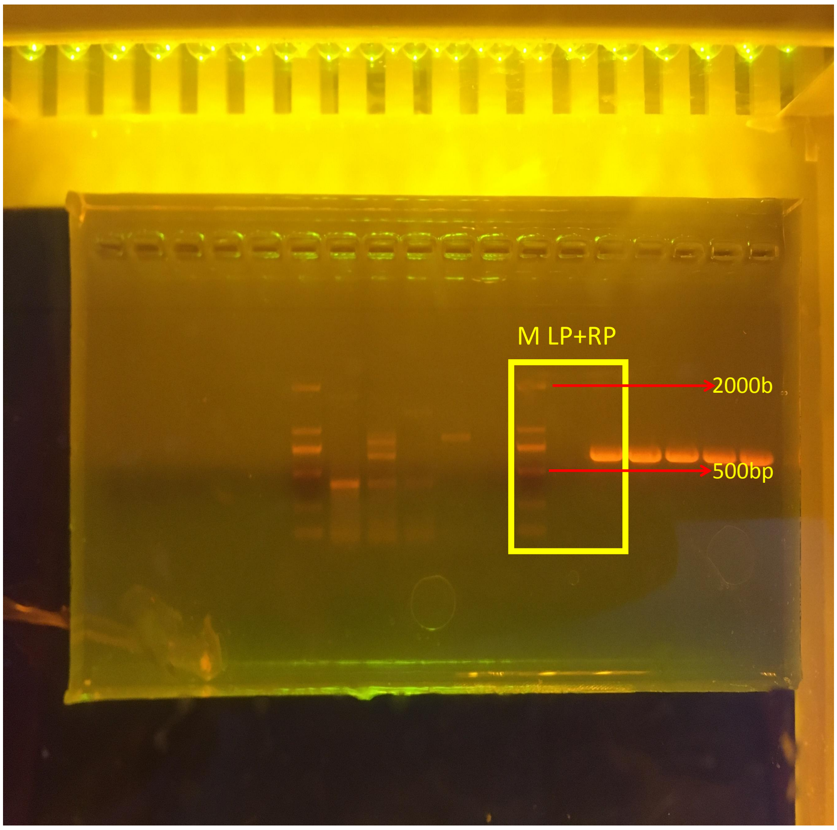

S5.*attbl43* half-length gels.

Note. The cropping figure（yellow box）used in the main body. M represents Mark, and the six band from top to bottom is 2000,1000,750,500,250,100bp, respectively.
